# Stuck in transit: A qualitative study of the transitional care needs of young people with epilepsy and juvenile idiopathic arthritis

**DOI:** 10.1177/13674935221074777

**Published:** 2022-03-02

**Authors:** Neil Wilson, Karen Whittaker, Janine Arnott, Lauren Burke, Michael W Beresford, Matthew Peak

**Affiliations:** 1 Faculty of Health and Care, University of Central Lancashire, Preston, UK; 2 Visiting Fellow, School of Nursing, 6723 University of Central Lancashire, Preston, UK; 3 Institute of Translational Medicine, 4591 University of Liverpool, Liverpool, UK; 4 200462 Alder Hey Children’s NHS Foundation Trust, Liverpool, UK

**Keywords:** Adolescent, arthritis juvenile, epilepsy, chronic disease, transition to adult care

## Abstract

Transition services for young people with long-term conditions often fall short. This qualitative study explored perspectives on service features that enable effective transition in epilepsy and juvenile idiopathic arthritis. Patients, parents, clinicians and service commissioners took part in semi-structured interviews (*n* = 18). Thematic analysis was used to identify key features, barriers and facilitators of effective transition across participant groups. Analysis led to the development of nine sub-themes which mapped to overarching domains of communication, capability, continuity and capacity. Findings include the need for age appropriate communication, the link between parental dependence, self-care and patient knowledge, the value of service integration for continuity and the impact of capacity on flexible and age appropriate transition services.

## Introduction

Advances in medical care and technology have enabled more young people (YP) with chronic medical conditions to survive into adulthood (Betz et al., 2021; Perrin et al., 2014). A corollary of this is that more YP with long-term conditions (LTC) undergo the transition from child to adult health care (McManus and White, 2017; Sabbagh et al., 2018).

Those with LTC can find transition difficult (Fegran et al., 2014) and are more at risk of poor health outcomes and disengagement with care following the move from children’s services (Coyne et al., 2019; Kelly and Wray, 2020; Lotstein et al., 2013). Over the last decade, transition services have been identified as a key area requiring significant improvement (Campbell et al., 2016) leading to development of several policy and guidance documents within the United Kingdom (e.g. Care Quality Commission, 2014; National Institute for Health and Care Excellence, 2016; Royal College of Nursing, 2013) and elsewhere (Foster et al., 2017; World Health Organization, 2015; Transitions Clinical Report Authoring Group, 2019).

The need for transitional care for patients with epilepsy is well established (Camfield et al., 2012; Lewis and Noyes, 2013). Epilepsy can have a significant psychosocial impact on YP during adolescence (Gray et al., 2017), with higher rates of mental health issues, and lower quality of life reported in those at transition age when compared to those without epilepsy (Healy et al., 2020). The expectations of self-management through transition means adolescent epilepsy patients are vulnerable to depression, poor self-confidence and self-esteem, and social maladjustment (Camfield and Camfield, 2014; Geerlings et al., 2015). Similarly, YP with chronic juvenile rheumatological conditions, despite progress in rheumatology transitional care (McDonagh and Farre, 2018), still experience difficulties over this period (Clemente et al., 2017; Foster et al., 2017; Sabbagh et al., 2018).

Beneficial transition service features, such as appropriate parental involvement, age-banded clinics and meeting adult teams in advance, have been associated with improved health outcomes in diabetes, autism and cerebral palsy (Colver et al., 2018a). This work has emphasized ‘developmentally appropriate health care’ (Farre et al., 2016; Rapley et al., 2019) that acknowledges the changing developmental needs of YP as they move through adolescence (Colver and Longwell, 2013), in order to provide flexible and personalized transition services (Hislop et al., 2016). A review of qualitative literature by Burke et al. (2018) also identified three key domains that underpinned service features and processes necessary for good transition in epilepsy and juvenile idiopathic arthritis (JIA): capability of the young person, communication and continuity of care between services.

The current study sought to empirically build on this work by exploring a range of stakeholder perspectives on transition in epilepsy and JIA. These diagnoses were chosen as we envisioned that learning from the study would be implemented in neurology and rheumatology services in participating hospitals. Coyne et al. (2019) noted that research incorporating the voices of YP and parents is limited when establishing empirical evidence of transition experiences. Multi-perspective and multi-systemic research is necessary (McDonagh and Farre, 2018) to help clarify and refine effective transition models (Betz et al., 2021). Although multi-stakeholder perspectives exist in studies (e.g. Crawford et al., 2021), few have also incorporated a commissioning^1^ perspective. Commissioning is under-researched yet can impact transition experiences (Kolehmainen et al., 2017; Maniatopoulos et al., 2018).

## Aim

To explore the perspectives of YP, parents, clinicians and commissioners regarding the key features supporting positive transition in epilepsy and JIA, including barriers and facilitators to good transition. Objectives included examining the domains suggested by Burke et al. (2018), and incorporating a wide range of perspectives, including before and after transition, from child and adult services, and from other organizational systems (commissioning).

## Method

### Ethics

Ethical approval was given by the University of Central Lancashire ethics review committee (ref: STEMH 415) and a National Health Service (NHS) ethical review committee (ref: 15/NW/0935). Research and Development permission was granted by participating hospitals. Informed written consent was obtained from all participants. No incentives were given for participation. The right to withdraw and protection of anonymity through use of participant codes was explained in information sheets and at the start of each interview. Data was collected and stored anonymously in accordance with University data management policy.

### Setting, study population and recruitment

Patients, parents and clinicians were recruited during 2017 from outpatient clinics in one children’s hospital, two general hospitals and a specialist hospital in the North West of England. Commissioners were recruited from an NHS Clinical Commissioning Group (CCG) in the North West of England.

*Young people*: YP were eligible for the study if they were between the age of 13 and 25, English speaking and were attending outpatient clinics for a diagnosis of epilepsy or JIA. Patients with severe learning difficulties or autism were not recruited as this might have altered transition services significantly. Where there was dual or complex diagnosis, advice was sought from treating clinicians regarding suitability for inclusion. Patients were purposively sampled to reflect sex, diagnosis and stage of the transition process (pre- or post-transfer). A member of the care team approached eligible candidates and offered them a study flyer and information sheet, and the researcher was on hand in clinics to answer questions. Interested families were followed-up by telephone call by the researcher to arrange an interview.

*Parents*: Parents were eligible for inclusion if they had children who met the patient inclusion criteria and attended outpatient clinics with their child. Parents and children were not included if the family had known safeguarding issues or where doing so was likely to cause distress, and this was advised by treating clinicians. Parents were recruited in the same manner as YP.

*Clinicians*: Clinical professionals were eligible for the study if they practiced in neurology or rheumatology teams in pediatric or adult services at the recruitment sites, and currently provided care for transitioning patients. Clinicians were recruited by direct email invitation.

*Commissioners*: Commissioners were eligible if they worked in a CCG in the North West of England. Commissioners were recruited through direct email invitation.

### Data collection

Participants took part in face-to-face semi-structured interviews. Interview guides were developed for each participant type, using open-ended questions and probes. Guides were informed by relevant literature and developed through consultation with experts in the research team, including qualitative researchers and health professionals in rheumatology and neurology. Two YP under 16 years checked YP guides for understanding, and these were amended based on feedback. Guides were used flexibly during interviews to accommodate participant views. Patient and parent questions were related to key areas of life with LTC, expectations of transition (pre-transfer families), experiences of and recommendations for transition (post-transfer families), and discussion of good transition and ideal outcomes. Interviews with YP and parents took place in the family home. Questions for clinicians and commissioners explored understanding of transition, professional experiences of managing transition and discussion of good transition outcomes. Clinicians and commissioners were interviewed at their work premises at a time convenient to them.

### Analysis

Interviews were audio recorded, transcribed verbatim and anonymized prior to analysis. Data were analysed using thematic analysis (Braun and Clarke, 2006) and supported by Nvivo 11 software (QSR International, USA). Transcripts were read multiple times and checked against interview audio, ensuring familiarity with the data and supporting researcher reflexivity. Two authors (NW and LB) open coded each transcript independently. Codes were discussed, refined and then grouped based on similarity, and assigned as sub-themes by the two coders. Disagreements were resolved with a third author (JA), who also supported review and refinement of sub-themes, and mapping using a table to the key domains of communication, continuity and capability. Sub-themes that did not map to these domains were analysed and grouped under new themes.

### Findings

The final sample included six patients, five parents, four clinicians and three commissioners of children’s services (Table 1). Interviews lasted between 30 and 60 minutes.

**Table 1.** Participant characteristics.

Participant	Characteristic	Epilepsy	JIA
Patient	Age (range) (years)	13–17	15–25
	Sex		
	Female	2	2
	Male	1	1
	Stage of transition		
	Pre-transfer	2	2
	Post-transfer	1	1
Parents	Relationship		
	Mother	1	3
	Father	1	0
Clinicians	Service		
	Pediatric	1	1
	Adult	1	1

Commissioners were non-condition specific and all three were child service commissioners.

*Note*. JIA: juvenile idiopathic arthritis.

Analysis identified nine sub-themes in the data, two of which mapped to an overarching domain of communication, two related to capability, three to continuity and two to capacity (Table 2).

**Table 2.** Themes and sub-themes.

1. Communication
Developmentally appropriate communication
Communication between services
2. Capability
Independence from parents
Knowledge and self-care
3. Continuity
Starting from scratch
Shared practices and joint working
Fragmentation of holistic care
4. Capacity
Flexible transition
Age appropriate environments

The interviews with YP and parents were conducted at different stages of the patient transition process (before and following transfer to adult services); hence, their data reflect actual or anticipated experiences (expectations).

### Communication

This theme refers to the communication needs of YP and families during transition, and to communication between clinicians in child and adult services.

#### Developmentally appropriate communication

Some YP prior to transition felt apprehensive at the prospect of dealing with adult clinicians, even explicitly invoking the need for age appropriate communication.

*‘I don’t want someone when I move to be like dead abrupt and like proper adulty…if it’s someone that is just too serious, like not for my age, then I feel a bit intimidated by them’*. (YP3, pre-transfer)

The shift in communication dynamics could be difficult for transferred patients, who felt unprepared for engaging with adult consultations. Age-inappropriate communication appeared to inhibit young people expressing themselves.

*‘That was the initial shock when you moved over, and I think when he did just talk directly to me it was overwhelming. I didn’t quite know what to say’.* (YP6, post-transfer)

Effective communication also included effective listening; one YP expressed frustration at not being heard in adult services, suggesting that clinicians can enable effective communication in YP by giving space for expression of them.

*‘I got upset at the beginning because they wouldn’t listen. They didn’t listen to what we’d been through….’ (*YP6, post transfer*).*

Parents also recognized that abrupt communication was a potential barrier to their child’s engagement with adult care – before they even reached the consultation room, for example, when making an appointment with administrative staff.

*‘I think it should be a specific person who is trained or who understands that this is a person who might not be confident on the phone. I think it should be one person who deals with a transition person. Whoever he spoke to put him off completely. So, he said then “you do it mum”’. (*Parent 1)

Overall clinicians recognized the pitfalls of age-inappropriate communication but tended to focus on the triadic communication dynamics in consultations involving YP and parents. One pediatrician was candid about their own difficulties navigating these shifting dynamics as their patients grew older.

*‘I think moving the consultation away towards more direct interaction with the young person perhaps without the parents, I do find that really difficult actually’*. (Clinician 1, pediatric)

However, clinicians also recognized the importance of non-verbal communication as a tool for engaging YP and managing triadic communication.

*‘I think it’s encouraging them to take part and make purposeful eye contact, “I can see your parents over there but I’m not going to focus on them…it’s you I want to hear from”’. (*Clinician 3, adult*)*

#### Communication between services

Communication between child and adult teams was mainly addressed by clinicians and commissioners, who recognized it as being fundamental to effective transition.

*‘The most important thing is the communication with paediatric services. If you have that communication, that trust of the teams and the capacity of going up and down with questions and discussions, that makes a whole difference’. (*Clinician 2, adult)

*‘For a particular service having good links and good communications because they are the ones that know individual patients and can say – “right this little lad’s going to need a bit more support, and this one you’ll find she’s fine, she’s very mature” can kind of prepare the receiving team for how to deal with an individual’*. (Commissioner 2)

However, parents also expressed frustration at the apparent lack of communication between services.

*‘The communication between the hospitals, they need to communicate’.* (Parent 1)

### Capability

This theme referred to the capability of patients to independently manage their own care, including medication. Often this was linked to parental involvement and the shift in responsibilities to the young person, as well as the developing knowledge of YP about their condition.

#### Independence from parents

YP prior to transfer offered mixed perspectives on the anticipated loss of parental involvement and the shift in responsibilities through transition, with some expressing concern, whilst others felt more empowered by the opportunity to be more engaged in their health care.

*‘I think it will be much harder because my mum and my family know what they are doing. So transferring I think it will be a bit different for me because I’ll have to make my own way to hospital and book appointments and stuff like that’.* (YP1, pre-transfer)

*‘It makes me feel like that I’m not like a little kid, I sort of have the responsibility and it’s my condition and I should have the right to choose if I want to try this or I don’t at the end of the day, rather than let your parents make the decision for you’. (*YP5, pre-transfer)

However, experiences of transferred patients attested to the potential distress caused when YP are ill-prepared for the sudden exclusion of parents.

*‘I’d just seen him say “no” to my mum and that was quite upsetting to see that your mum’s not part of it…’* (YP6, post-transfer)

This divergence of perspective was mirrored by parents, some of whom resisted the marginalization of their involvement, whilst others recognized it as a necessity for developing independence in their child.

*‘I think there needs to be that acknowledgement that parents still need to be heavily involved. Parents should still have the right to speak to consultants, to speak to nurses’. (*Parent 3*)*

*‘All of a sudden it’s daunting for a parent, it really is, and not within your remit…the parents - while he’s still living in this house I need to know what’s going on’. (*Parent 5*)*

*‘It is a good thing because she’s getting older, so I should be just in the back anyway you know listening, because she will have to go by herself sometimes won’t she?’* (Parent 4)

Overall, clinicians suggested that there was a balance to be struck in parental involvement, and in particular in respecting the patient’s growing need for privacy and naturally changing boundaries with parents.

*‘They should be part of the process but at the same time we do need to have some time separate from them as well… There are a number of things that it’s good to have confidential consultations with the young person’.* (Clinician 4, pediatric)

#### Knowledge and self-care

In preparing YP for transition, clinicians saw a key indicator of their capability to be their knowledge of the condition and self-care. In some instances, clinicians saw parental dependence as a barrier to developing knowledge.

*‘What we’re so struck by is they get to say sixteen and they are going to move on and some of the young people don’t even know what tablets they are on, what type of epilepsy they have, what epilepsy is, because they’ve just been tagging along to clinic and it’s their parents who do everything for them’.* (Clinician 4, pediatric)

*‘You can’t make an informed decision on whether or not you are going to get bladdered the night before…if you don’t have that knowledge to make those independent decisions…It’s about making sure that their knowledge base is ok and that there are no gaps, there’s no new questions that are arising’.* (Clinician 3, adult)

The link between parental involvement and knowledge was illustrated by one epilepsy patient.

*‘I find I’ve learnt a lot more from the transition going in on my own about my medication…You kind of have to know it, it forces you to know it’.* (YP5, pre-transfer)

However, the importance of early preparation was emphasized to ensure successful self-care.

*‘Start to introduce self-care, self-management earlier maybe and can test out – are they able to do that*?’ (Commissioner 2)

### Continuity

Continuity refers to the degree of co-ordination between child and adult services to ensure a seamless transition and to prevent YP disengaging from their care. It includes the recognition of patient journey and the avoidance of abrupt changes to established care trajectories.

#### Starting from scratch

YP often saw their time in child services as a long, sometimes hard-won process of optimizing their treatment plan. There was clear anticipatory anxiety that moving to adult services could undo this work. Familiarity with patients, or their case history, in advance of transfer meant that YP did not have to re-tread old ground.

*‘If I went to (place) and all my treatment and all the progress that I’d done has been lost…that would be my worst scenario.’* (YP2, pre-transfer)

*‘Just that they know me and it’s not like I have to tell them like everything, because they already know it’.* (YP3, pre-transfer)

Parents echoed the same sentiments, expressing fear and frustration at the possibility of having to start again in adult services.

*‘We are finally on the correct road and my fear is that we suddenly get handed over to an adult doctor and they go – “oh no, I don’t agree with that” – and they change that and before you know it she’s back to square one’.* (Parent 3)

*‘I’m sick of explaining to everybody, you know, how she is and stuff. I’d like for it to be known and for someone before they even meet her to get to know her’.* (Parent 2)

#### Shared practices and joint working

A key facilitator of continuity through the transition process was the inclusion of integrated transition clinics. YP agreed that meeting the adult team in advance of transfer helped develop trust and confidence.

*‘My most important thing would be that I met the team before anything is put in place… because if not I feel like I don’t trust them….’* (YP2, pre-transfer)

*‘Once you’ve had that familiar face I think you are more open to talk… Bit by bit you do get more confident… if you are meeting them beforehand I think they will feel a bit more comfortable’.* (YP6, post-transfer)

Overall, clinicians agreed that trust and engagement were most effectively established when meeting pediatric and adult teams together.

*‘If you just meet the adult nurses – “who’s this, can I trust her?” Whereas if you meet her together with the paediatric nurse you’ve known for so long it’s like – “well if they work together they must be good people”… We’ve had some examples of young people who initially didn’t engage with the adult nurse at all but when they came to this joint clinic all of a sudden, they started to engage’.* (Clinician 4, pediatric)

#### Fragmentation of holistic care

All commissioners addressed issues around the organization of adult services, particularly transition in complex cases involving multiple health conditions. Such cases were highlighted as at particular risk of care fragmentation.

*‘They might transition to the care of four specialties, so who takes the lead, who co-ordinates their care, who takes the child’.* (Commissioner 1)

*‘Adult services are all built on single specialties and it doesn’t work’*. (Commissioner 3)

Conversely, the strategic avoidance of fragmentation could have the effect of delaying transfer altogether.

*‘Many young people stay under the care of paediatric services for much longer than they should because adult services aren’t properly constructed, commissioned, to accept them into their care at the moment’.* (Commissioner 1)

Organizational and systemic characteristics of adult services were often cited as barriers to transition. The fragmentation of care was illustrated by clinicians, who also noted that mental health services were at particular risk during the period of transition.

*‘He then had his regular epilepsy care transitioned but his [condition] they kept back as if he was in like three compartments, you know like your arm goes first, your leg goes next and then your torso goes in the middle’.* (Clinician 3, adult)

*‘Psychology as an example, between sixteen and eighteen there is not a very good service for psychology…it’s impossible, nobody wants to see you’.* (Clinician 4, pediatric)

One parent felt abandoned by psychological services and felt burdened when coordinating disparate services as their child approached transition.

*‘She had to be eighteen, so we were just sort of left again. She’s one of those who is at that age where she is either too young for certain things or she’s too old for others’.* (Parent 2)

*‘What I needed… the hospital, the doctors, the mental health team all work together… I had loads of appointments for different things, but nothing actually gels together…. It’s always been me trying to find something to get her through’.* (Parent 2)

### Capacity

This theme concerns the impact of inadequate service capacity and resources to ensure tailored and adolescent-friendly transition.

#### Flexible transition

Overall clinicians and commissioners emphasized the need for flexibility in the timing of transfer, and for transition that is tailored to the young person’s needs. However, this appeared to be dictated by capacity issues.

*‘I think the reason why services often aren’t flexible is because of capacity, if there’s a capacity issue people always throw service specifications at you, you know – “my service goes to sixteen, bang, leave…” they get completely rigid which means that there are a number of patients falling in-between the systems and that is dreadful’.* (Clinician 4, pediatric)

*‘A really important component of transition is that it’s not textbook, we have an available pathway, but we base that decision on their individual needs and how they might respond to that’.* (Commissioner 2)

#### Adolescent appropriate environments

Capacity was also reflected in the suitability of waiting areas. There was an overall consensus that these environments were not designed to be adolescent or transition friendly. YP at different stages of transition reported feeling uncomfortable in their respective waiting areas of hospitals, illustrating the changing developmental needs of transition age YP.

*‘It felt awkward for me waiting in that room because there was five, six, seven year olds and then there was me, and it’s just like – right this is a bit awkward, I don’t feel quite comfortable’.* (YP5, pre-transfer)

*‘I just felt a bit uncomfortable because I was like young compared to everyone there’.* (YP4, post-transfer)

Clinicians and commissioners recognized this and advocated greater capacity for adolescent-friendly environments.

*‘I think they often find the move to this new environment away from a paediatric environment difficult. Maybe a case mix within a waiting area is going to look and feel very different’.* (Clinician 1, pediatric)

*‘It’s all about age appropriate services… Ideally there should be paediatric, adolescent and adult clinics separately. If I had the money, I would convert one of the wards to an adolescent ward’.* (Clinician 4, pediatric)

*‘They are just in the wrong environment…they are not age appropriate facilities’.* (Commissioner 3)

## Discussion

The aim of this study was to qualitatively explore the service features, barriers and facilitators of good transition in epilepsy and JIA and to identify how findings support the transition processes identified by Burke et al. (2018). A wide range of perspectives were gathered from different stakeholders involved in transition services to achieve this. The incorporation of commissioning services in the study allowed a multi-systemic as well as a multi-perspective approach.

YP and parents expressed the need for sensitive and age appropriate communication from clinicians and administrative staff, the latter of whom may be the first point of contact for YP before they meet clinical teams. Indeed, pre-transfer participants anticipated less personable encounters with staff in adult services (Coyne et al. (2019). YP were sometimes unprepared for shifting communication dynamics in adult services and felt frustrated when they did not feel listened to or understood, which can act as a barrier to receiving personalized care (Hislop et al., 2016; Renedo et al., 2019), and deny the expertise patients and their families have in their condition (Williams et al., 2021).

Some clinicians also struggled with shifting communication dynamics. This could prevent inclusion of YP in care consultations and decisions, and hamper independence. Research suggests that YP wish to be more involved in consultations in ways that suit them (Van Staa, 2010). Dedicated training in the communication needs of YP could therefore help professionals to encourage YP to be more engaged in their care in ways that are developmentally appropriate (Rapley et al., 2019).

Findings illustrated that the capability of YP in managing self-care was influenced by parental involvement, supporting previous work (Van Staa et al., 2011a; Sabbagh et al., 2018) and by knowledge of their condition and medication. An interplay of parental involvement and knowledge often underpinned YP and parental anxieties around patients becoming more responsible for their care, because of previous dependence on parents to know things. However, as illustrated by this study, parents often find transition difficult partly because they are required to relinquish and devolve care responsibilities, yet wish to remain involved (Coyne et al., 2019; Crawford et al., 2021; Shaw et al., 2021).

Nevertheless, one epilepsy patient attributed his increasing knowledge to the absence of parents in consultations. Indeed, the balance of patient and parental knowledge swings as care responsibilities shift through transition (Gray et al., 2017). Moreover, this expansion of knowledge appeared empowering, supporting findings that when parental withdrawal is well managed YP can see this as an opportunity for independence (Coyne et al., 2019; Crawford et al., 2021; Heath et al., 2017). Planned parental withdrawal can also allow YP to be more directly involved in discussions and decisions around their care (Van Staa, 2010) and potentially improve health outcomes (Colver et al., 2018a).

A key finding of this study was the fear of change to established care as a result of differing clinical opinions in adult teams, and of ‘starting again’, which loomed large in the concerns of patients and families. This could compound the anticipated loss of trusted relationships with child teams and undermine the development of trust in new teams (Coyne et al., 2019; Tuchman et al., 2008). However, the value of co-ordination between child and adult teams through joint clinics was emphasized by clinical staff. Such integration was suggested to foster YP familiarity, confidence and trust in adult teams, supporting work that recognizes that such service features are essential for effective transition and continuity of care (e.g. Foster et al., 2017).

Continuity of care was identified by commissioners as being at particular risk for those with multiple or complex conditions, and for whom holistic care can fragment into multiple transitions (Williams et al., 2021). This was largely attributed to the organization and commissioning of adult services as ‘single specialty’ and unequipped for holistic care, suggesting that better integration at commissioning levels are also needed (Kolehmainen et al., 2017; Maniapoloupos et al., 2018).

Finally, Foster et al. (2017) suggested that the absence of resources should not dictate the timing of transfer, yet resource issues are a frequently reported barrier to transition (McDonagh and Farre, 2018). This study found that capacity issues could lead to delayed transfer, particularly in complex cases, and also hampered clinician’s ability to provide flexible and personalized transition based on the needs of patients (Hislop et al., 2016). Moreover, the lack of adolescent appropriate hospital environments and facilities reflected capacity issues, despite their importance being well established (Colver et al., 2018a; Foster et al., 2017; Renedo et al., 2019). Without suitable settings, YP can feel alienated or intimidated in the places intended to care for them (Crawford et al., 2021).

### Strengths and limitations

A strength of this study was its support of the need for multi-systemic and multi-perspective research (McDonagh and Farre, 2018), by bringing together a range of perspectives across different organizational systems: YP and parents before and following transition, child and adult services, and commissioning organisations. Our patient sample age range was broad in recognition of transition as an extended process encompassing different developmental stages (Rapley et al., 2019). Limitations include the small number of overall participants and of post-transfer patients, preventing insights to broader patient experience. The absence of adult service commissioning perspectives limits what can be said around integrated commissioning (Maniatopoulos et al., 2018), and potentially biases findings towards child commissioning services.

### Implications for practice

The study suggests that the topic of transition should be included within training and education programmes for those working with transition age patients (clinical and non-clinical staff), giving particular attention to young person development, age appropriate communication and recognising the lived experience of YP and families. There are also important implications for those designing services around integration of structures that enable communication across child and adult professional teams, and sensitivity to the different levels of young person maturity as they transition towards adulthood. Greater integration of commissioning services is also needed to reduce fragmentation of care.

## Conclusion

This study highlighted the importance of recognising the unique perspectives of multiple stakeholders when considering effective transition care. Vital service features broadly relate to a framework of communication, capability, continuity of care and capacity of services. Communication often underpins and supports patient capability and service continuity; hence, interventions to tackle specific elements of transition would do well to recognize how often they are interlinked. Greater communication and integration of child and adult services, and developmentally appropriate care can help to ensure that YP become capable custodians of their own health, as parents devolve responsibilities.

## Note

1. Commissioning is the process of assessing, planning, purchasing, and monitoring health care service provision for a population. In England this is carried out by Clinical Commissioning Groups and the NHS at local, regional, and national levels.
